# HDAC2 Partially Contributes to the Differential Nephrotoxicity Induced by AA-I, AA-IVa, or AL-I

**DOI:** 10.3390/ph19101612

**Published:** 2026-10-10

**Authors:** Yuan Wang, Zhong Xian, Jing Meng, Chunying Li, Yong Zhao, Yan Yi, Jingzhuo Tian, Xiaoxin Gao, Chen Pan, Jiayin Han, Yushi Zhang, Suyan Liu, Chenyue Liu, Xingwen Wang, Feng Gao, Yuan Yu, Shuang Cui, Meiting Liu, Lianmei Wang, Aihua Liang

**Affiliations:** 1 State Key Laboratory for Quality Ensurance and Sustainable Use of Dao-Di Herbs, Institute of Chinese Materia Medica, China Academy of Chinese Medical Sciences, Beijing 100700, China; 2 Institute of Traditional Chinese Medicine, Tianjin University of Traditional Chinese Medicine, Tianjin 301617, China; 3 Beijing Key Laboratory of Psychoactive Substances Discovery and Control in Chinese Herbal Medicines, Institute of Chinese Materia Medica, China Academy of Chinese Medical Sciences, Beijing 100700, China; 4 Key Laboratory of TCM Safety Risk Assessment and Translation, Institute of Chinese Materia Medica, China Academy of Chinese Medical Sciences, Beijing 100700, China; 5 Beijing Institute for Drug Control, Beijing 102206, China; 6 College of Pharmacy, Guizhou University of Traditional Chinese Medicine, Guiyang 550025, China

**Keywords:** aristolochic acid analogs, nephrotoxicity, fibrosis, apoptosis, HDAC2

## Abstract

**Background:** ASARI RADIX ET RHIZOMA (Xixin), extensively utilized in clinical settings, contains aristolochic acid analogs (AAAs), primarily AA-IVa, with minor quantities of AL-I and negligible levels of AA-I. AA-I mediates aristolochic acid-induced nephropathy; therefore, the safety of Xixin is controversial. The toxicity of AAAs is varied, and the mechanisms are ambiguous. **Objective:** To compare the toxicity of AA-I, AA-IVa, and AL-I toward renal tubular epithelial cells and explain the molecular causes of such. **Materials and Methods:** C57BL/6 mice were administered AA-I, AA-IVa, or AL-I for 24 weeks; renal toxicity was assessed via survival and histopathology. In vitro, AAAs toxicity was evaluated in human renal tubular epithelial cells (HK-2) by high-content Hoechst 33342/PI apoptotic tests and fibrosis marker quantification. Transcriptomics revealed alterations in genes and pathways induced by AAAs. Click chemistry-based activity-based protein profiling, molecular docking, surface plasmon resonance, enzyme activity assays, and gene knockdown identified the AAAs’ direct targets. **Results:** AA-I induced dose-dependent mortality, severe kidney injury, and fibrosis; AL-I showed less toxicity, and AA-IVa was not discernibly toxic in mice. In vitro, AA-I prompted apoptosis and fibrosis in HK-2 via transcriptional modification; other AAAs exhibited diminished effects. AA-I, AL-I and AA-IVa showed stable binding ability to HDAC2; AA-I and AL-I enhanced HDAC2 expression; and AA-I facilitated HDAC2 nuclear translocation and enzymatic activity. AA-I, unlike AL-I and AA-IVa, augmented MYC expression, disturbed the BCL2/BAX equilibrium, activated Caspase-7, and triggered apoptosis and pro-fibrotic transformation. **Conclusions:** AA-I induces apoptosis and fibrosis through HDAC2, and HDAC2 partially contributes to the various toxicities of AA-I, AA-IVa, and AL-I. This study provides fundamental evidence for the safety evaluation of Xixin.

## 1. Introduction

Aristolochic acid analogs (AAAs) are a category of naturally small-molecule phenanthrene compounds primarily found in plants of the genera *Aristolochia* and *Asarum* (family *Aristolochiaceae*). In 2012, the International Agency for Research on Cancer (IARC) categorized aristolochic acids (AAs) and the plants containing AAs as Group I carcinogens due to their capacity to cause urinary tract cancers and aristolochic acid nephropathy (AAN) [1]. Recently, investigators found that the toxicity of diverse types of AAAs and related medicinal plants were varied significantly [2,3]. Aristolochic acid I (AA-I), aristolochic acid II (AA-II), and traditional Chinese remedies containing them were the culprits of AAN [4]. Aristolochic acid IVa (AA-IVa) exhibits structural similarity to AA-I, distinguished solely by the presence of an extra hydroxyl group. AA-I generates DNA adducts in vivo; AA-IVa produces DNA adducts in vitro but not in vivo [5]. Moreover, the acute toxicity in mice and cytotoxic efficacies of these two substances vary by factors of tens to hundreds [2,6]; nevertheless, the fundamental mechanisms remain ambiguous.

ASARI RADIX ET RHIZOMA (Xixin) is the sole species of *Aristolochiaceae* featured in the 2025 edition of the Chinese Pharmacopoeia (CHP), which contains *Asarum heterotropoides* Fr. Schmidt var. *mandshuricum* Maxim. Kitag. (Bei Xixin), *Asarum sieboldii* Miq. var. *seoulense* Nakai (Hancheng Xixin), and *Asarum sieboldii* Miq. (Hua Xixin). There are 44 Chinese patent medicines in CHP containing Xixin, with a suggested dosage of 1–3 g of Xixin decoction per day [7]. Xixin contains AAAs including AA-IVa (48.49–121.03 μg/g), aristolactam I (AL-I, 14.04–43.75 μg/g), and AA-I (0.4–2.4 μg/g), and it is devoid of AA-II or AA-IIIa [2,8,9]. Prolonged toxicity tests in mice indicate that oral treatment of Bei Xixin decoction at 100 times the clinical dose for 12 or 24 weeks, followed by an 8-week withdrawal period, does not result in substantial gastric, hepatic, renal, or bladder impairment [8]. Xixin has exhibited clear therapeutic efficacy and distinct benefits in the clinical management of respiratory ailments, digestive disorders, and rheumatoid arthritis [10]. Clinical investigations have demonstrated that Xixin does not elevate the risk of chronic kidney disease or uroepithelial cancers (UCs) [11,12]. In various Asian regions, including Japan and South Korea, Xixin-containing formulations such as Xiaoqinglong decoction, Mahuang Fuzi Xixin decoction, Xixin powder/decoction, and Danggui Sini decoction remain prevalent for the treatment of colds, rhinitis, headaches, and toothaches [13,14]. Nonetheless, the safety of Xixin is still controversial due to the content of AAAs.

The defining pathological hallmarks of AAN comprise infiltration of inflammatory cells, tubular atrophy, and interstitial fibrosis [15]. AA-I induces oxidative stress and mitochondrial dysfunction in renal tubular epithelial cells, resulting in apoptosis, structural disruption of the tubules, and ultimately functional impairment. Simultaneously, AA-I initiates Smad-independent pathways and promotes autocrine TGF-β signaling, initially leading to renal interstitial fibrosis [16]. Nevertheless, the mechanism by which AA-I selectively damages renal tubular epithelial cells, along with the associated molecular targets and the initiation of downstream apoptotic and pro-fibrotic signaling cascades, remains ambiguous. It is also pivotal for us to distinguish the relationship between structure and toxic effect among AAAs.

In this study, we utilized the AA-I small-molecule probe (Prode 1) in conjunction with activity-based protein profiling and click chemistry technology (ABPP-CC) to identify toxic targets, and HDAC2 was identified as the target for AA-I. AA-I accelerated renal tubular epithelial cell damage and fibrosis by upregulating HDAC2 expression, nuclear localization, and enzyme activity, as well as partially contributing to the varied toxicity among AA-I, AA-IVa, and AL-I. This study provides fundamental evidence for the safety evaluation of herbs containing AAAs, such as Xixin.

## 2. Results

### 2.1. Chronic Nephrotoxicity of AA-IVa and AL-I Is Significantly Lower than That of AA-I

**Figure 1:**
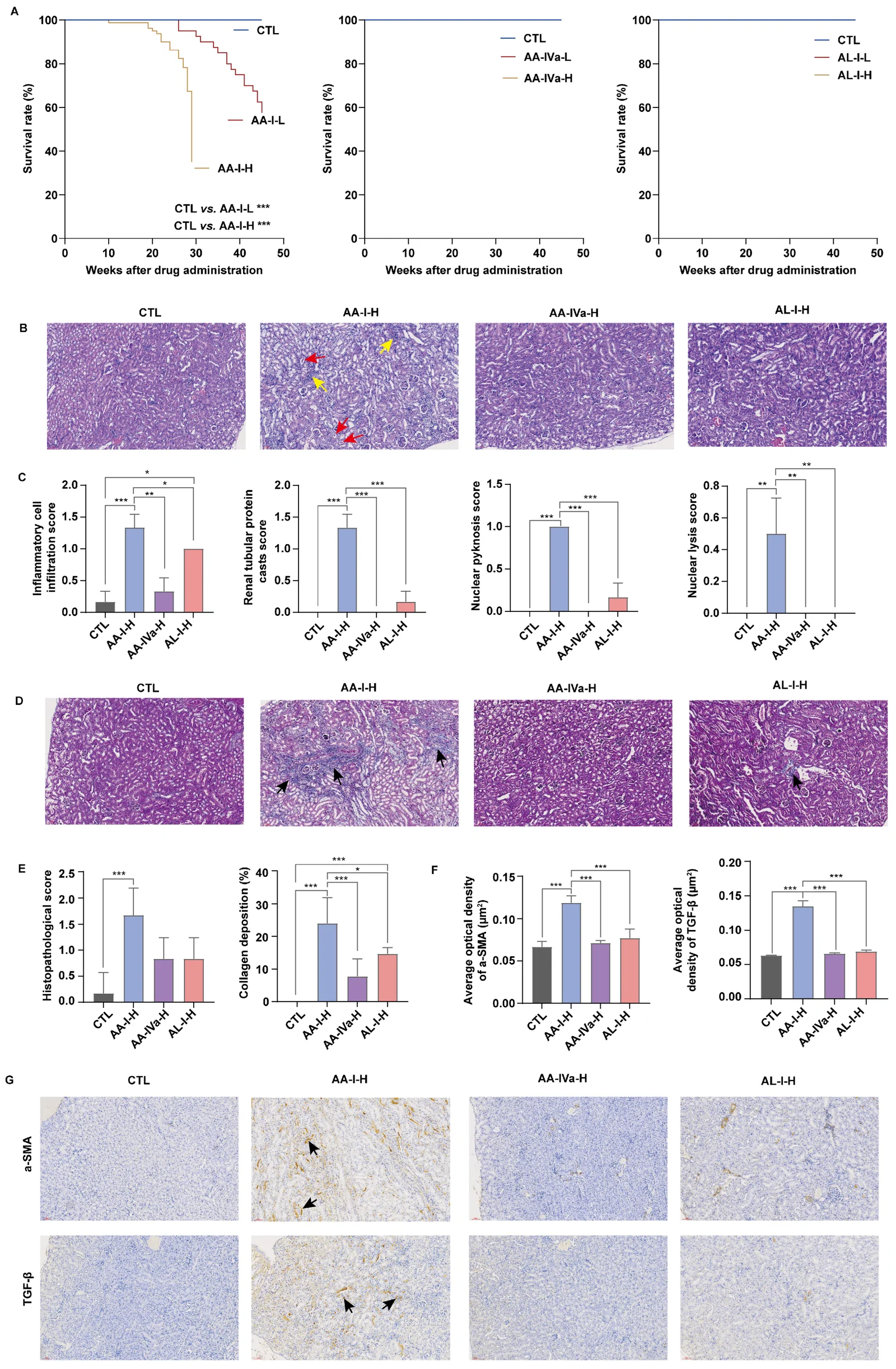
Toxicity evaluation of aristolochic acid analogs, including aristolochic acid I (AA-I), aristolochic acid IVa (AA-IVa), and aristolactam I (AL-I). (**A**). Kaplan–Meier curves show the survival of mice after AAA administration for 24 weeks, followed by 21 weeks of medication cessation; *n* = 30–80. (**B**,**C**). Hematoxylin and eosin (HE) staining (**B**) and histological assessments (**C**) of mouse renal tissues subjected to 10 mg/kg AAA treatment. In (**B**), yellow arrowheads indicate prominent inflammatory cell infiltration, nuclear pyknosis, and nuclear lysis; red arrows indicate obvious casts. (**D**,**E**). Masson’s trichrome staining (**D**), fibrosis assessments, and quantitative (**E**) investigation of collagen deposition in mouse renal tissues subjected to 10 mg/kg AAA treatment. In (**D**), arrowheads indicate renal fibrosis. (**F**,**G**). Semiquantitative (**F**) and immunohistochemistry (IHC) assessment of renal fibrosis markers α-SMA and TGF-β (**G**) in mouse renal tissues. In (**G**), arrowheads indicate antibody positive staining. *n* = 6. * *p* < 0.05; ** *p* < 0.01; *** *p* < 0.001.

This study was performed to assess the chronic toxicity variations among AA-I, AA-IVa, and AL-I. Mice received oral administration of AA-I, AA-IVa, or AL-I for 24 weeks and were observed every day until 45 weeks; kidney tissues were harvested for histological analysis at the endpoint of the study. AA-I significantly induced mouse mortality in a time- and dosage-dependent way in contrast to CTL mice (Figure 1A). In addition, none of the mice died after AA-IVa or AL-I administration (Figure 1A).

Renal HE histopathology indicated that the score of lymphocytic inflammatory cell infiltration, renal tubular protein casts, and nuclear pyknosis and lysis of renal tubular epithelial cells increased significantly in AA-I-H mice, and lymphocytic inflammatory cell infiltration was evidently elevated in AL-I-H mice in contrast to CTL mice (Figure 1B,C). Additionally, the scores of lymphocytic inflammatory cell infiltration, renal tubular protein casts, and nuclear pyknosis and lysis of renal tubular epithelial cells decreased dramatically in AL-I-H and AA-IVa-H mice in contrast to AA-I-H mice (Figure 1B,C).

Compared to CTL mice, AA-I-L mice exhibited only minor localized lesions, sporadic protein casts, punctate interstitial inflammatory cell infiltration, and infrequent pyknosis, without evident karyolysis of renal tubular epithelial cells. AL-I-L mice exhibited only minimal inflammatory infiltration, and no discernible lesions were observed in AA-IVa-L mice (Figure S1A,B).

Masson’s trichrome staining revealed that AA-I administration at both 10 mg/kg and 1 mg/kg led to substantial interstitial fibrous growth and collagen accumulation in renal tissues (Figure 1D,E and Figure S1C,D). AL-I at the same two doses did not clearly cause interstitial fibrous growth; instead, it merely increased collagen deposition (Figure 1D,E and Figure S1C,D). Furthermore, the severity of collagen deposition in the AA-I group was markedly greater than those in the AL-I or AA-IVa groups (Figure 1D,E and Figure S1C,D). In addition, substantial interstitial fibrous cells were not observed in the AA-IVa-treated mice (Figure 1D,E and Figure S1C,D).

TGF-β and α-SMA serve as molecular indicators of renal fibrosis [16], and their expression levels were evaluated in mouse kidney sections in AAA-treated mice via IHC analysis. TGF-β and α-SMA increased in AA-I-H mice compared to CTL, AL-I-H, and AA-IVa-H mice; although they slightly increased in AA-IVa-H and AL-I-H mice, the alterations were not dramatically in contrast to CTL mice (Figure 1F,G). In addition, the expressions of TGF-β and α-SMA were not obviously different among all low-dose AAA-treated cohorts of mice (Figure S1E,F). The results demonstrate that AA-I enhances the upregulation of fibrotic markers in a dose-dependent fashion. The extent of kidney damage and pro-fibrotic risk caused by AA-IVa and AL-I was markedly less than that caused by AA-I.

### 2.2. AA-IVa and AL-I Elicit Markedly Fewer Fibrotic and Apoptotic Responses in Renal Tubular Epithelial Cells Compared to AA-I

**Figure 2:**
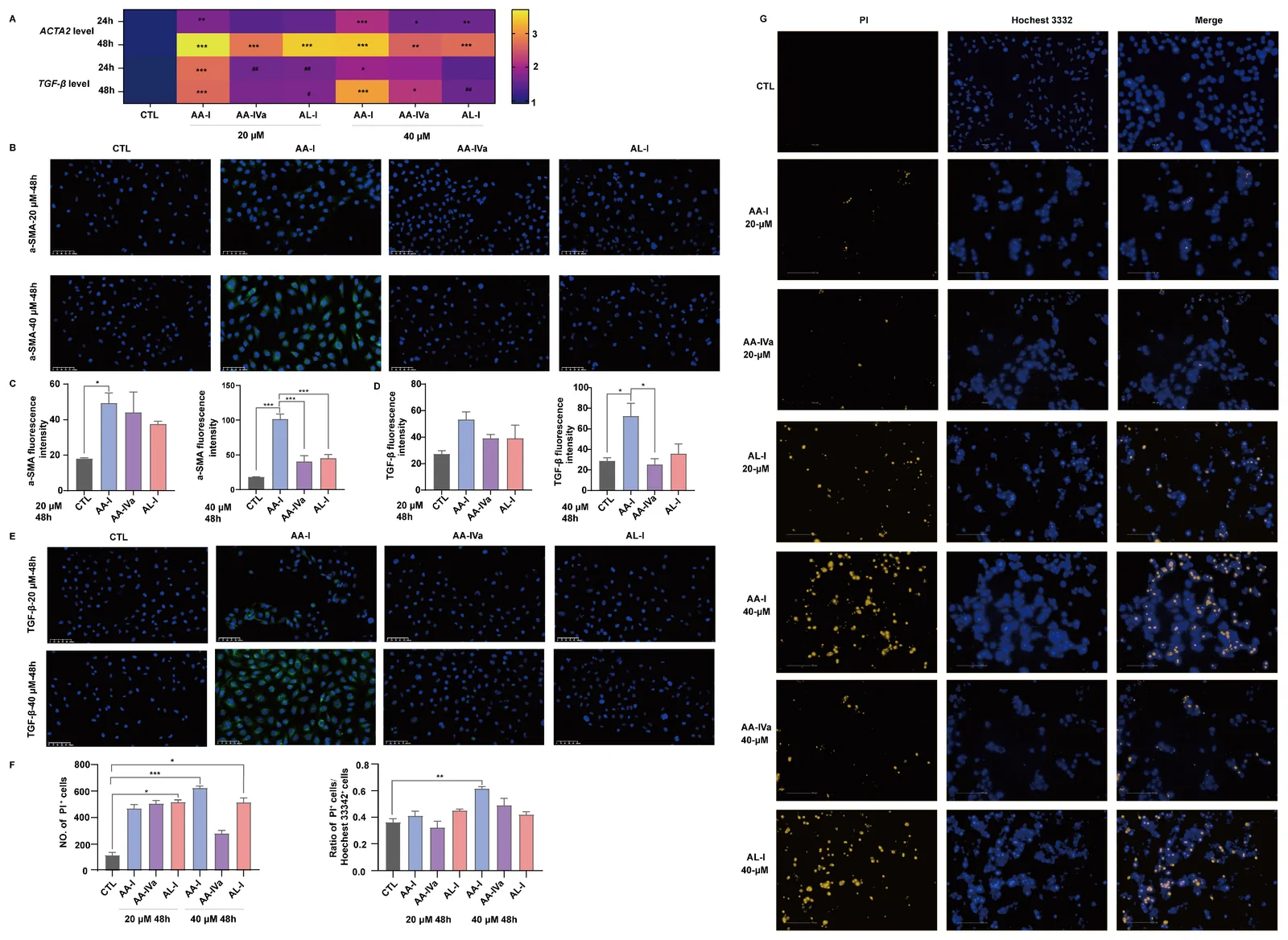
AA-I promotes fibrosis and apoptosis in human renal tubular epithelial (HK-2) cells, while AA-IVa and AL-I have diminished effects. (**A**). Quantitative PCR of *ACTA2* and *TGF-β* in HK-2 cells treated with AAA; * *p* < 0.05; ** *p* < 0.01; *** *p* < 0.001 vs. Control (CTL). ^#^ *p* < 0.05; ^##^ *p* < 0.01 vs. AA-I. (**B**–**E**) Immunofluorescence (IF) staining for α-SMA (**B**) and its quantification * *p* < 0.05; *** *p* < 0.001 (**C**), and quantification * *p* < 0.05 (**D**) and IF staining for TGF-β (**E**) in HK-2 cells treated with AAA; DAPI (blue), nuclei; green, target protein. (**F**,**G**). Quantification * *p* < 0.05; ** *p* < 0.01; *** *p* < 0.001 (**F**) and high-content (**G**) examination of apoptosis following treatment with AAA for 48 h. *n* = 3.

HK-2 cells were treated with 20 μM or 40 μM of AAA for 24 hours (24 h) or 48 hours (48 h), and the expressions of α-SMA and TGF-β at the mRNA and protein levels were assessed by qPCR and IF, respectively. The mRNA levels of *ACTA2* (which encodes α-SMA) and *TGF-β* (which encodes TGF-β) were markedly elevated after AA-I treatment compared to the medium control group (Figure 2A). AL-I (20 μM treatment for 24 h or 48 h, and 40 μM treatment for 48 h) markedly reduced *TGF-β* expression in comparison to the AA-I-treated group. AA-IVa markedly upregulated *TGF-β* expression compared to CTL cells at 40 μM for 48 h, and downregulated *TGF-β* expression compared to AA-I-treated cells at 20 μM for 24 h (Figure 2A). IF analysis demonstrated that treatment with 20 μM or 40 μM AA-I for 48 h considerably elevated α-SMA protein levels, and treatment with 40 μM AA-I for 48 h significantly increased TGF-β protein levels in contrast to CTL cells (Figure 2B–E). Following 40 μM of AA-IVa or AL-I treatment for 48 h, α-SMA expression levels were dramatically reduced compared to the AA-I-treated group (Figure 2B,C). Additionally, TGF-β levels dramatically decreased in the 40 μM of AA-IVa for 48 h treatment in contrast to the AA-I-treated group (Figure 2D,E). The results demonstrate that AA-I effectively produces a fibrotic phenotypic transformation in HK-2 cells, while AA-IVa and AL-I do not significantly activate fibrotic signaling in these cells.

The apoptosis of renal tubular epithelial cells is a significant factor in the advancement of kidney damage and fibrosis [17]. HK-2 cells were subjected to treatment with 20 μM or 40 μM of AAA for durations of 48 h, and apoptosis was evaluated using Hoechst 33342/PI double labeling in conjunction with high-content imaging. Following treatment with AA-I (40 μM), there was a considerable increase in both the absolute number and the fraction of late apoptotic/necrotic cells, demonstrating a pronounced pro-apoptotic effect of high-concentration AA-I (Figure 2F,G). Under uniform conditions, AL-I (20 μM or 40 μM) resulted in an elevation of PI^+^ cells (Figure 2F,G); however, the late apoptotic/necrotic ratio exhibited no significant variation. The data indicate that AA-I significantly promotes apoptosis in HK-2 cells, and AL-I has a minor pro-apoptotic effect but it is weaker than that of AA-I; conversely, AA-IVa has no discernible apoptosis-inducing activity.

### 2.3. RNA Sequencing Uncovers AA-I-Specific Pronounced Pro-Fibrotic Regulatory Characteristics

**Figure 3:**
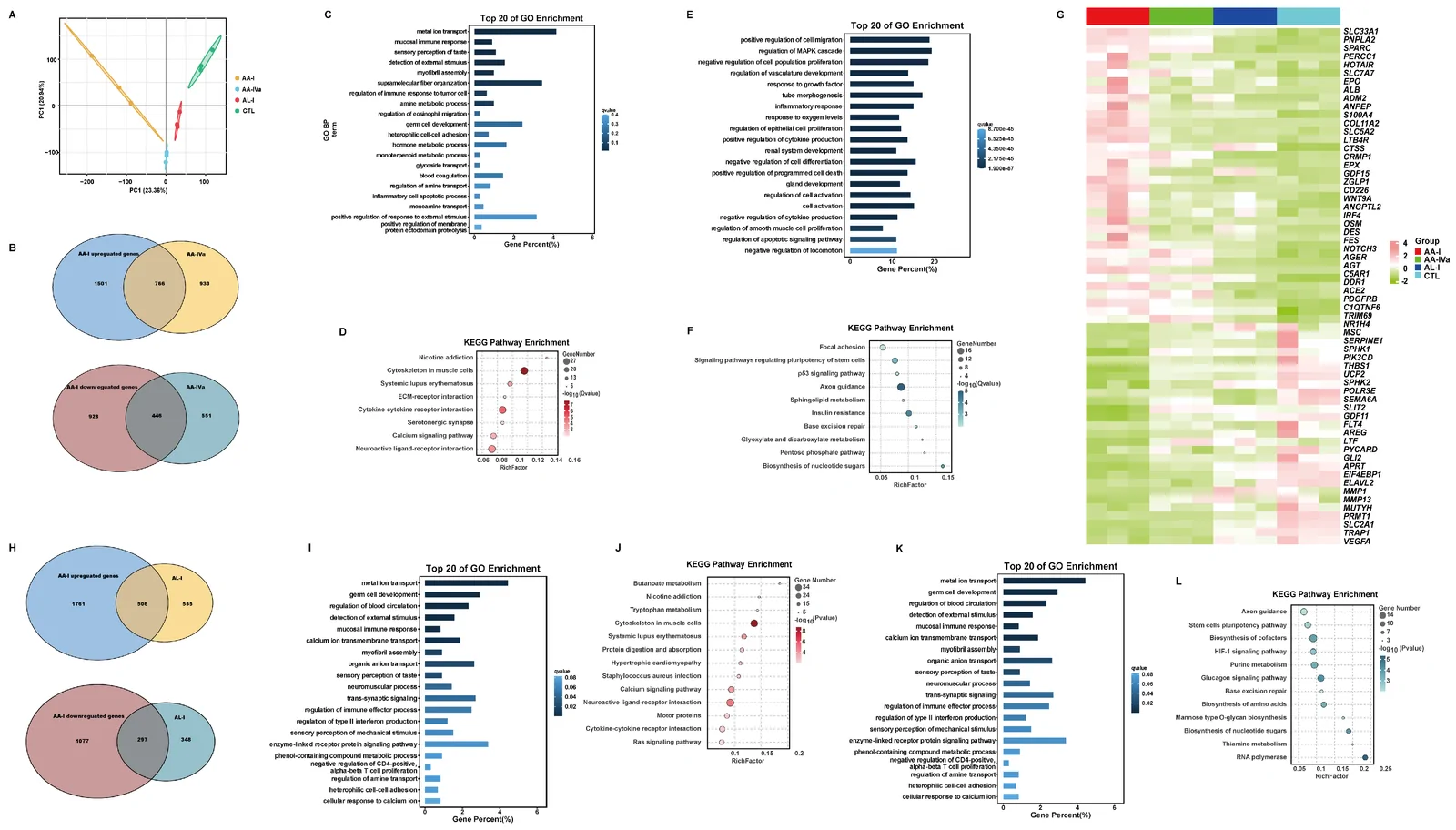
Transcriptomic analysis reveals unique pro-fibrotic regulatory features of AA-I in HK-2 cells. (**A**,**B**). Principal component analysis of all samples (**A**), and comparison between AA-I and AA-IVa (**B**). (**C**–**F**). Gene Ontology—Biological Process (GO-BP) and Kyoto Encyclopedia of Genes and Genomes (KEGG) pathway enrichment analyses with upregulated genes (**C**,**D**) and downregulated genes (**E**,**F**), with a comparison between AA-I and AA-IVa. (**G**). Heatmap of genes related to renal fibrosis between AA-I and either AA-IVa or AL-I. (**H**). Comparison between AA-I and AA-IVa. (**I**–**L**). GO-BP and KEGG pathway enrichment analyses with upregulated genes (**I**,**J**) and downregulated genes (**K**,**L**), with a comparison between AA-I and AL-I. *n* = 3.

RNA sequencing was conducted to evaluate the impact of AA-I, AA-IVa, and AL-I on the transcriptome of HK-2 cells. Principal component analysis demonstrated that the transcriptional regulation pattern of the AA-I-treated group displayed significant clustering disparities compared to the CTL, AA-IVa, and AL-I groups (Figure 3A). Analysis of DEGs revealed that AA-I modified the expression of 3641 genes, comprising 2267 upregulated and 1374 downregulated genes (Figure 3B). AA-IVa affected 2696 genes, with 1699 upregulated and 997 downregulated (Figure 3B). The 1501 genes specifically upregulated by AA-I were predominantly enriched in pathways associated with the cytoskeleton and motor function (e.g., myofibril assembly and cytoskeleton in muscle cells), immune inflammatory responses (e.g., cytokine–cytokine receptor interactions), neurosignaling (e.g., neuroactive ligand–receptor interactions), and remodeling of the extracellular matrix microenvironment (e.g., ECM–receptor interactions) (Figure 3C,D). The 928 genes specifically downregulated by AA-I were predominantly enriched in pathways related to cell adhesion and migration (e.g., positive regulation of cell migration and focal adhesion), metabolic processes (e.g., pentose phosphate pathway), regulation of stem cell pluripotency (e.g., signaling pathways regulating pluripotency of stem cells), and DNA damage repair (e.g., p53 signaling pathway) (Figure 3E,F). Further examination of the 45 principal renal fibrosis genes specifically modulated by AA-I revealed that the upregulated genes were implicated in metabolic regulation (e.g., *ADM2* and *GDF15*), extracellular matrix remodeling (e.g., *SPARC* and *COL11A2*), and immune inflammation (e.g., *IRF4* and *OSM*), while the downregulated genes were linked to cell proliferation and survival (e.g., *PIK3CD* and *THBS1*), extracellular matrix degradation (e.g., *SERPINE1* and *MMP13*), and DNA repair (e.g., *MUTYH*) (Figure 3G). Additionally, AL-I modified the expression of 1706 genes, comprising 1061 upregulated and 645 downregulated (Figure 3H). AA-I activation pathways particularly upregulated 1761 genes associated with cytoskeletal and motor functions, neurosignaling, and immunological inflammation (Figure 3I,J). The 1077 genes specifically downregulated by AA-I demonstrated inhibition of pathways related to genetic information processing (e.g., RNA polymerase), biosynthesis and metabolism (e.g., purine metabolism), and cell fate control (e.g., HIF-1 signaling pathway) (Figure 3K,L). Heatmap analysis of 56 essential renal fibrosis genes indicated that AA-I treatment upregulated pro-fibrotic, inflammation-enhancing, and tissue-remodeling genes, including *NOTCH3*, *PDGFRB*, and *C5AR1*, while downregulating genes such as *PIK3CD*, *GLI2*, and *VEGFA*, potentially compromising cellular repair mechanisms, antioxidant stress responses, and pro-angiogenic activities (Figure 3G). The transcriptomic analysis reveals that, in contrast to AA-IVa and AL-I, AA-I significantly activates pathways related to cytoskeletal rearrangement, inflammatory responses, and dysregulated metabolism, while concurrently inhibiting genes associated with cyto-protection, extracellular matrix homeostasis, and DNA repair.

### 2.4. ABPP-CC Designates HDAC2 as a Target of AA-I Toxicity

**Figure 4:**
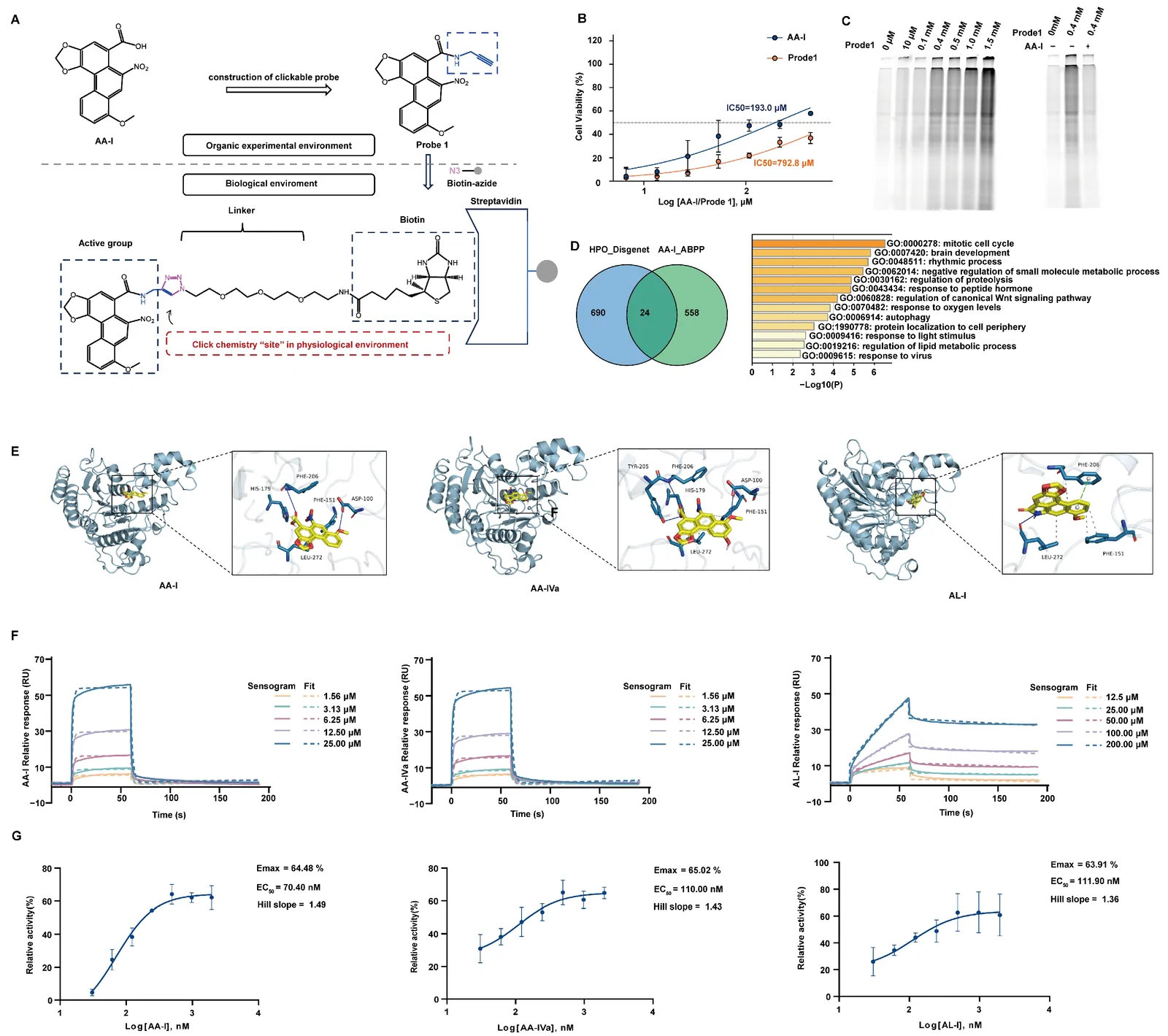
Activity-based protein profiling combined with click chemistry (ABPP-CC) designated HDAC2 as a direct target of AA-I. (**A**). Diagram illustrating the production of the AA-I probe (Probe 1). (**B**). Cell Counting Kit-8 (CCK-8) assay of Probe 1 and AA-I in HK-2 cells after 24 h treatment. (**C**). Optimization of Probe 1 labeling concentration and AA-I competitive binding test. (**D**). GO-BP enrichment analysis of AA-I-targeted proteins linked to renal fibrosis using mass spectrometry. (**E**–**G**). Analyses of molecular docking (**E**), protein purification and surface plasmon resonance (SPR) (**F**), and stimulation of HDAC2 enzymatic activity by AA-I, AA-IVa, and AL-I (**G**), revealing their binding modalities with and functional effects on HDAC2. *n* = 3.

In this study, we utilized an ABPP-CC technique to identify direct molecular targets of AA-I-induced nephrotoxicity. A probe of AA-I, modified with an alkyne, referred to as Probe 1, was initially synthesized (Figure 4A). Cell viability experiments demonstrated that the half-maximal inhibitory doses (IC_50_) of Probe 1 and AA-I in HK-2 cells were 792.8 μM and 193.0 μM, respectively, suggesting that Probe 1 modification did not markedly enhance AA-I cytotoxicity (Figure 4B). At optimal gradient concentrations, 0.4 mM Probe 1 demonstrated elevated signal-to-noise ratios in protein labeling, and competitive assays validated that Probe 1’s binding to target proteins was both highly specific and competitive (Figure 4C).

A total of 582 potential target proteins were first discovered from the pull-down complexes using label-free mass spectrometry. By analyzing renal fibrosis-related targets, 24 common genes were identified by comparison with the HPO and DISGENET databases. GO—BP enrichment analysis indicated that the largest functional cluster was primarily associated with “mitotic cell cycle”, comprising 17 genes, which represents 70.83%, implying that AA-I may influence its effects by disrupting cell cycle regulatory networks (Figure 4D).

Molecular docking simulations were conducted for principal potential targets within this pathway, revealing that HDAC2 exhibited stable binding to AA-I, AA-IVa, and AL-I, with binding free energies of −8.27 kcal/mol, −8.88 kcal/mol, and −9.57 kcal/mol, respectively (Figure 4E). HDAC2 is essential for epigenetic regulation, cell cycle management, and apoptosis. HDAC2 elevation is observed in chronic kidney disease models and accelerates fibrotic progression by enhancing apoptosis and inflammatory responses [18]. Binding mode analysis demonstrated that AA-I engages with critical residues of HDAC2 (PHE-206 and ASP-100) through two robust conventional hydrogen bonds, further reinforced by hydrophobic interactions and a salt bridge with HIS-179. AA-IVa establishes three hydrogen connections with PHE-206, ASP-100, and TYR-205, while AL-I mostly interacts via a hydrogen bond with LEU-272 and π-π stacking with PHE-206 (Figure 4E).

The recombinant human HDAC2 protein was purified (purity > 95%) and verified by SDS-PAGE in conjunction with Western blotting with a distinct band at 58 kDa (Figure S2). The KD values for AA-I, AA-IVa, and AL-I were measured at 56.60 μM, 68.80 μM, and 34.50 μM, respectively, with the kd values being 0.49 (1/s), 0.45 (1/s), and 0.0018 (1/s). This data indicates that AA-I and AL-I bind to HDAC2 tightly, while AA-IVa exhibits the lowest affinity. The ka values for AA-I, AA-IVa, and AL-I were 0.0087 (1/Ms), 0.0066 (1/Ms), and 0.53 (1/Ms), respectively, indicating that AL-I binds to HDAC2 most rapidly (Figure 4F).

Furthermore, HDAC2 enzyme activity was detected after AAA treatment. AA-I, AA-IVa, and AL-I exhibited similar maximal activation levels of HDAC2 (Emax: 63.91–65.02%). AA-I demonstrated the highest activation potency for HDAC2 (EC_50_ = 70.4 nM, Hill slope = 1.49), whereas AA-IVa (EC_50_ = 110.0 nM, Hill slope = 1.43) and AL-I (EC_50_ = 111.9 nM, Hill slope = 1.36) exhibited lower activation potency (Figure 4G).

### 2.5. HDAC2 Is a Crucial Regulator Facilitating AA-I-Induced Damage and Fibrosis in Renal Tubular Epithelial Cells

**Figure 5:**
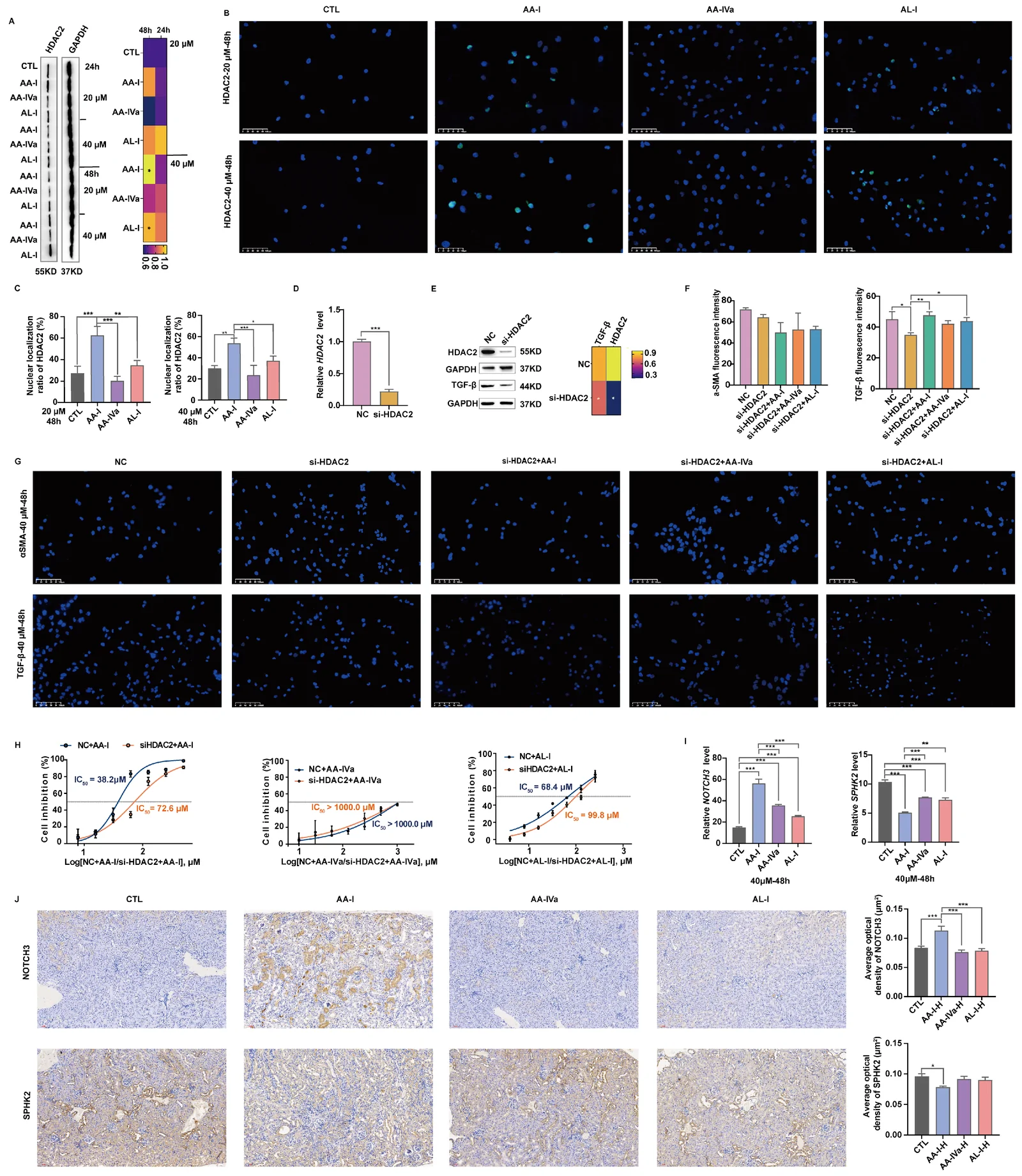
HDAC2 facilitates AA-I-induced damage and fibrosis in HK-2. (**A**). HDAC2 expression detection by western blot and quantification; * *p* < 0.05 vs. CTL. (**B**,**C**). IF (**B**) and quantification (**C**) were performed to detect HDAC2 in HK-2 cells treated with AAA. DAPI (blue), nuclei; green, target protein; * *p* < 0.05; ** *p* < 0.01; *** *p* < 0.001. (**D**,**E**). HDAC2 knockdown in HK-2 cells, qPCR (**D**), and Western blot and quantification (**E**) validation following siHDAC2 transfection in HK-2 cells; * *p* < 0.05; *** *p* < 0.001 vs. NC. (**F**,**G**). Quantification * *p* < 0.05; ** *p* < 0.01 (**F**) and IF (**G**) analysis of TGF-β and α-SMA in HK-2 cells treated by the indicated media. DAPI (blue), nuclei; green, target protein. (**H**). Cell viability analysis in HK-2 cells treated by the indicated media. (**I**). *NOTCH3* and *SPHK2* mRNA expression in HK-2 cells treated with AAA. *n* = 3. ** *p* < 0.01; *** *p* < 0.001. (**J**). IHC and quantification of NOTCH3 and SPHK2 in mouse renal tissues following 10 mg/kg AAA exposure. Brown indicates positive staining. *n* = 6. * *p* < 0.05; *** *p* < 0.001.

Treatment of HK-2 cells with 40 μM AA-I or AL-I for 48 h resulted in the upregulation of HDAC2 protein expression, whereas AA-IVa did not affect HDAC2 protein levels (Figure 5A). Following the treatment of HK-2 cells with 20 μM or 40 μM AAA for 48 h, nuclear HDAC2 expression was markedly increased in AA-I-treated cells; HDAC2 was predominantly localized in the cytoplasm in AL-I- and AA-IVa-treated cells (Figure 5B,C). Knockdown of *HDAC2* in HK-2 cells markedly diminished TGF-β1 expression levels (Figure 5D,E). Moreover, following the treatment of HDAC2-knockdown cells with 40 μM concentrations of the three AAAs for 48 h, there was no observed elevation of α-SMA or TGF-β1 protein, and their expression levels did not significantly differ from the control group (Figure 5F,G). CCK8 cytotoxicity experiments revealed that, post-HDAC2 knockdown, the IC_50_ of AA-I increased from 38.2 μM to 72.6 μM, while that of AL-I increased from 68.4 μM to 99.8 μM, demonstrating that HDAC2 influences the associated cytotoxicity. The IC_50_ of AA-IVa consistently exceeded 1000.0 μM, both prior and subsequent to HDAC2 knockdown (Figure 5H). The integration of the transcriptome DEG data (Figure 3G) revealed that AA-I particularly modulated the expression of HDAC2-related genes [19,20]; *NOTCH3* was elevated, while *SPHK2* was downregulated in the AA-I-treated cells, with these alterations being much more pronounced than those caused by AL-I or AA-IVa (Figure 5I). The results were validated in vivo; IHC revealed that, in kidney tissue from mice given 10 mg/kg AA-I via gavage for 24 weeks, NOTCH3 expression was markedly elevated, and SPHK2 expression was diminished in renal tissues, while no such alterations were noted in the AL-I or AA-IVa treatment groups (Figure 5J).

### 2.6. AA-I Induces Apoptosis via the HDAC2–MYC Pathway

**Figure 6:**
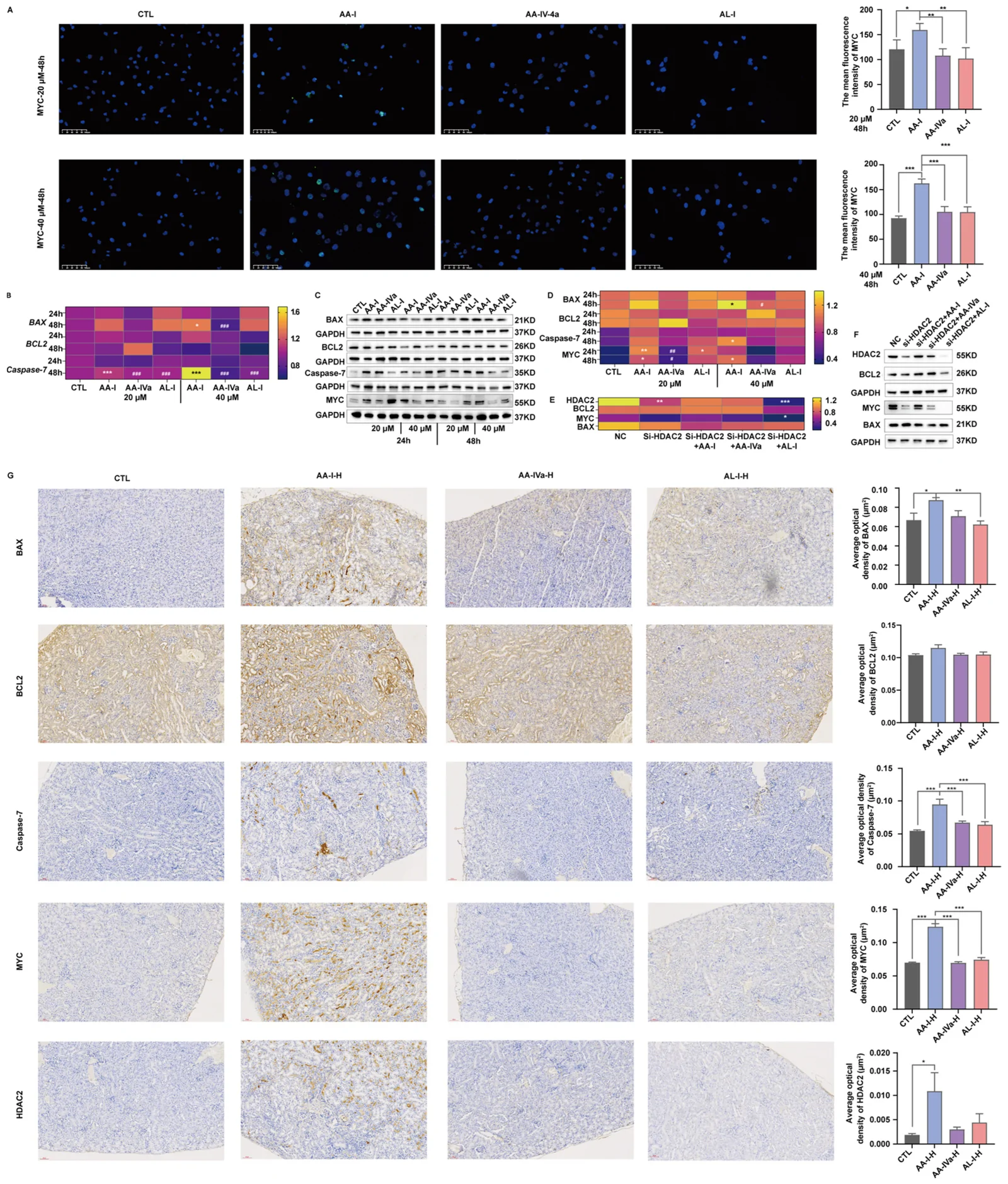
AA-I activates the mitochondrial-dependent apoptotic pathway in HK-2 cells through HDAC2-MYC. (**A**). MYC cellular localization and expression following treatment with AAA by IF; DAPI (blue), nuclei; green, target protein; * *p* < 0.05; ** *p* < 0.01; *** *p* < 0.001. (**B**,**D**). qPCR (**B**), Western blot (**C**), and quantification (**D**) analyses of the indicated protein in HK-2 cells treated with AAA; * *p* < 0.05; ** *p* < 0.01; *** *p* < 0.001 vs. CTL. ^#^ *p* < 0.05; ^##^ *p* < 0.01; ^###^ *p* < 0.001 vs. AA-I. (**E**,**F**). Quantification (**E**) and Western blot (**F**) analysis of the indicated protein in HDAC2 knockdown HK-2 cells. * *p* < 0.05; ** *p* < 0.01; *** *p* < 0.001 vs. NC. *n* = 3. (**G**). IHC and quantification analysis of BAX, BCL2, Caspase-7, MYC and HDAC2 protein in mouse renal tissues following AAA exposure, brown indicates positive staining. * *p* < 0.05; ** *p* < 0.01; *** *p* < 0.001. *n* = 6.

HDAC2 is a crucial regulator of apoptosis and can influence the transcription factor MYC [21]. MYC promotes fibrosis by facilitating epithelial cell damage [22]. Treatment with 20 μM or 40 μM AA-I for 48 h enhanced MYC protein expression and nucleus translocation, while no similar effect was noted in the AA-IVa or AL-I treatment groups (Figure 6A). MYC is a crucial regulator of the mitochondrion-dependent apoptotic pathway. BAX and BCL2, both constituents of the BCL2 protein family, preserve mitochondrial membrane integrity through their equilibrium. MYC influences this mechanism by regulating the expression of BAX and BCL2 [21]. In the 20 μM and 40 μM AAA treatments, AA-I administration for 24 h did not significantly alter *BAX* gene expression in HK-2 cells; however, after 48 h of treatment, 40 μM AA-I enhanced *BAX* gene expression, demonstrating a significant difference from the AA-IVa group (Figure 6B). Additionally, AA-IVa and AL-I did not influence *BAX* gene expression (Figure 6B). The expression of the anti-apoptotic gene *BCL2* was unaffected by any of the three AAAs (Figure 6B). The protein expression patterns of BAX and BCL2 aligned with the gene expression levels, suggesting that AA-I disturbs the BAX/BCL2 equilibrium at 40 μM concentrations, consequently facilitating apoptosis (Figure 6C,D). Caspase-7, a pivotal executor of mitochondrion-dependent apoptosis, demonstrated no significant alteration in gene expression following 24 h of treatment with 20 μM or 40 μM AA-I. However, *Caspase-7* mRNA expression increased after 48 h of AA-I treatment, significantly surpassing levels observed in the CTL, AA-IVa, and AL-I groups (Figure 6B). Treatment with 40 μM AA-I for 48 h dramatically increased Caspase-7 expression at the protein level (Figure 6C,D). Conversely, AA-IVa and AL-I did not elicit elevation of Caspase-7 gene or protein expression following 24 h or 48 h of treatment at concentrations of 20 μM or 40 μM (Figure 6B–D). The MYC protein in HK-2 cells exhibited significant upregulation following treatment with 20 μM AA-I for 24 h or 48 h, as well as with 40 μM AA-I for 48 h (Figure 6C,D), displaying a pattern nearly identical to that of BAX and Caspase-7 protein expression, thereby further indicating that AA-I may affect the expression of mitochondrial-dependent apoptosis-related proteins. siHDAC2 was administered to HK-2 cells for 24 h, and then they were exposed to 40 μM AA-I, AA-IVa, or AL-I for 48 h. The findings indicate that the elevations in HDAC2 and MYC protein levels, together with the deregulation of BAX/BCL2 protein expression caused by AA-I, were mitigated (Figure 6E,F). Furthermore, in the AL-I-treated cohort, HDAC2 and MYC proteins exhibited considerable downregulation, indicating a potential inhibitory action (Figure 6E,F).

IHC data confirmed the activation of this route by AA-I in mouse kidney tissues. AA-I induced the elevation of HDAC2, MYC, BAX, and Caspase-7 in renal tissue sections, but BCL2 displayed no discernible alteration among all cohorts of mice (Figure 6G). These results indicate that AA-I could affect the expression of MYC and the mitochondrial apoptosis-related protein BAX through HDAC2, thereby potentially playing a role in renal tubular epithelial cell injury, whereas no similar effects were observed for AA-IVa or AL-I (Figure 6G).

## 3. Discussion

This study demonstrated the toxicity variety of AAAs including AA-I, AA-IVa and AL-I. AA-I induced dose-dependent mortality, significant renal damage, and fibrosis in mice; AL-I showed reduced toxicity, while AA-IVa exhibited no apparent toxicity. AA-I specifically targeted HDAC2 to promote apoptosis and fibrosis; HDAC2 was partially responsible for the toxicity disparities across AA-I, AA-IVa, and AL-I. This study establishes a crucial foundation for the risk evaluation of herbal medications containing AAAs, such as Xixin.

### 3.1. The Toxicity of AAAs Varies

Over 238 AAAs have been discovered [23], primarily categorized into two classes, namely, AAs and aristolactams (ALs) [8]. AA-I is metabolically activated in vivo; the nitro group within its structure can covalently attach to DNA, resulting in persistent adducts, which is a fundamental molecular mechanism contributing to its carcinogenicity and renal damage [24]. The DNA adducts exhibit remarkable stability [25], exert sex-specific toxicological effects [26], and may be identified in patient tissues decades post-exposure [25]. They trigger mutations in critical genes such as TP53 and FGFR3, resulting in a substantially elevated risk of kidney damage and UC [25]. AL-I is a reductive metabolite of AA-I; its overall toxicity was significantly less than that of AA-I. A 24-week regimen of AL-I administration in mice did not lead to mortality, acute renal injury, or signs of mutagenicity/tumorigenicity; however, delayed renal injury was observed after 50-week cessation [27]. Consistently, our work demonstrates that AL-I induced minor kidney injury and fibrin deposition in mice; nevertheless, its nephrotoxicity is considerably less severe than that of AA-I. Xue et al. indicated that AL-I failed to induce substantial renal tubular necrosis or fibrosis in vivo [28]. AA-IVa is a nitrophenanthroic acid and possesses a hydroxyl group at C_3_, which is absent in AA-I. This distinction facilitates the metabolism of AA-IVa into a more polar dihydroxyl molecule, enhancing its excretion and hence markedly diminishing its toxicity [2,29]. AA-IVa does not generate DNA adducts in vivo and yields negative results in the bone marrow micronucleus test [5]. A six-month chronic toxicity evaluation with mice administered 1 or 10 mg/kg of AA-IVa, followed by a 12-month recovery phase observation, demonstrated no substantial mutagenic or carcinogenic risk induced by AA-IVa [6]. AA-I produces nephrotoxicity in mice by activating the p38-STAT3-S100A11 signaling pathway, but AA-IVa does not activate this pathway [30].

### 3.2. AAAs’ Variations in Content and Composition Directly Influence the Toxicity Risk of the Chinese Herbal Medicines

AA-I is found in significant concentrations in *Aristolochia manshuriensis* Kom. (Guanmutong) (about 2.85 mg/g) [31]. In rats, intragastric administration of a water decoction of Guanmutong at dosages of 50 or 30 g/kg/day for 7 days, or at 20 g/kg/day for 15 days, followed by one month of observation, resulted in acute tubular necrosis [32]. The roots of *Aristolochia debilis* Siebold & Zucc. (Qingmuxiang) and *Aristolochia fangchi* Y.C.Wu ex L.D.Chow & S.M.Hwang (Guangfangji), both of which contain AA-I, with concentrations of 1.19–4.71 mg/g or 0.64–4.23 mg/g, respectively [33], have been banned in numerous countries due to their considerable toxicity risk. At a cumulative AAAs dosage of 3 mg/kg, *Aristolochia cinnabarina* C.Y.Cheng & J.L.Wu (Zhusalian) and the fruit of *Aristolochia debilis* Siebold & Zucc. (Madouling), both possessing elevated AA-I concentrations (2.00–5.91 mg/g and 0.35–0.72 mg/g, respectively), elicited renal interstitial fibrosis and stomach damage [3]. Under identical conditions, the stem and leaf of *Aristolochia debilis* Siebold & Zucc. (Tianxianteng), with its predominant AAA component being AA-IVa (AA-IVa is 0.12–0.35 mg/g and AA-I is 7.00–36.00 μg/g), elicited very mild toxic effects [3]. AA-IVa (48.49 μg/g), AL-I (14.0 μg/g), and AA-I (2.18 μg/g) were detected in Bei Xixin, while AA-II and AA-IIIa were absent [8]. Long-term intragastric administration of Xixin water decoction, even at doses significantly surpassing clinical use (up to a 100-fold clinical dose, 5.0 g/kg), did not result in notable injury to mice [7] or rats [34]. The Ames test, chromosomal aberration test, and micronucleus test indicated that the aqueous extract of Xixin root is non-genotoxic [34].

### 3.3. HDAC2 as a Target Mediates AA-I-Induced Renal Fibrosis, and Partially Explains the Toxicity Variations Among AA-I, AA-IVa, and AL-I

In this research, transcriptomics was performed to delineate the AA-I-specific toxicological profile. Despite the variety of enhanced or diminished pathways, they were unified around tubular damage and fibrosis [35,36], the fundamental pathological characteristic of AAN. Consequently, ABPP was utilized to identify AA-I directly binding target proteins. A total of 582 potential target proteins were discovered. Then we analyzed renal fibrosis-related targets. A total of 24 common genes were identified by comparison with the HPO and DISGENET databases. GO-BP enrichment analysis indicated that the largest functional cluster was primarily associated with the “mitotic cell cycle”, comprising 17 genes. Therefore, molecular docking and cell experiments were conducted to screen the targeted proteins for distinct toxicity of AAAs, and finally HDAC2 was chosen as the target protein.

Histone deacetylases (HDACs) involve at least ten HDAC isoforms, primarily class I (HDAC1/2/3/8) and class II (HDAC4/5/6/9), among others [37]. HDAC inhibitor chidamide, a selective inhibitor of HDAC1/2/3/10, reduces renal fibrosis by inhibiting the PI3K/AKT pathway [38]. HDAC1/2/3 expressions were markedly increased in renal tubular epithelial cells in a mouse model of aristolochic acid nephropathy [39,40]. Nevertheless, there is currently little proof that these HDACs are AAAs’ direct targets, and it is still unclear how distinct AAAs regulate HDAC-mediated toxicity. Our work shows that AA-I binds to HDAC2, accelerates its protein expression, nuclear translocation, and enzyme activity, and induces apoptosis and fibrosis in renal tubular epithelial cells and murine kidney tissue. We found that AA-I and AL-I bind to HDAC2 tightly, and AL-I binds to HDAC2 most rapidly among these AAAs; however, AL-I and AA-IVa exhibited a weaker ability to promote HDAC2 nuclear translocation and enzyme activity than AA-I. HDAC2 functions as an epigenetic transcriptional repressor, with its nuclear protein expression and the level of histone deacetylation being critical markers of its activation [41]. Therefore, AL-I and AA-IVa exhibited diminished effectiveness in modulating the HDAC2-associated pathway. Among these AA-I-responsive proteins, AA-I, but not AA-IVa or AL-I, upregulated NOTCH3 and downregulated SPHK2 in HK-2 cells. HDAC2 knockdown reinstated the protein levels of MYC, BAX, TGF-β, and α-SMA in HK-2 cells subjected to AA-I. Consistently, NOTCH3 overexpression stimulates extracellular matrix deposition by triggering the NF-κB pathway [19]. SPHK2 downregulation relieves its suppression of HDAC2 enzymatic activity [20]. Additionally, HDAC2 was reported to activate fibroblasts by regulating pro-fibrotic proteins and the TGF-β signaling pathway [42], and to regulate mitochondrion-dependent apoptosis by BCL2 family proteins and the Caspase pathway [43]. However, the mechanism by which AA-I influences MYC and its subsequent mitochondrial dysfunction resulting in renal tubular epithelial cell injury necessitates additional experimental validation.

We noted that the knockdown of HDAC2 elevated the half-maximal inhibitory concentration (IC_50_) of AA-I from 38.2 µM to 72.6 µM, signifying a substantial decrease in cytotoxicity that was, however, not entirely eradicated, implying that HDAC2 plays a partial role in AA-I-induced nephrotoxicity. This could be attributed to the fact that the systemic nephrotoxicity of AA-I is not exclusively reliant on its direct engagement with a single harmful target. The in vivo distribution of AA-I is affected by elements such as saturation absorption, metabolic conversion, and bioavailability, demonstrating significant nonlinear kinetic properties [26]. The renal uptake activity mediated by OAT1/OAT3 significantly influences AA-I-induced nephrotoxicity in vivo; the inhibition of OAT transporter activity might diminish renal exposure to AA-I, consequently mitigating its nephrotoxic effects [44]. Given the influence of the in vivo disposition of AA-I, the relationship between its accumulation in kidney tissues and its toxicity mechanism remains to be further investigated. Moreover, our RNA-sequencing data indicate the potential engagement of supplementary processes. Notably, unlike AA-IVa and AL-I, AA-I distinctly influenced pathways unequivocally linked to nephrotoxicity, such as neurosignaling (serotonergic synapse) and DNA damage response (p53 signaling pathway). These pathways are regarded as not entirely dependent on HDAC2 [45,46], and the associated hazards necessitate additional scrutiny.

This research has several limitations. Firstly, our clarification of the causal link between HDAC2 and MYC-driven, mitochondrion-dependent apoptosis is insufficient and requires additional confirmation. Secondly, HDAC2 only partially explains the variations in toxicity among the three drugs, and additional pathways warrant investigation. Moreover, the stimulating impacts of AAAs on HDAC2 cannot dismiss the likelihood of nonspecific binding and allosteric influences, and their mechanisms of action require additional exploration. Nevertheless, the findings indicate that HDAC2 plays a partial role in the toxicity variations between AA-I and AA-IVa/AL-I, thereby offering new evidence for the significantly divergent toxicity mechanisms of these three AAAs.

## 4. Materials and Methods

### 4.1. Antibodies and Reagents

Antibodies targeting α-SMA, TGF-β, HDAC2, NOTCH3, SPHK2, MYC, Caspase-7, BCL2, BAX, and GAPDH were procured from Proteintech (Wuhan, China). AA-I, AA-IVa, and AL-I (purity > 98%) were acquired from Chengdu Push Bio-Technology (Chengdu, China). DMEM/F12 culture media, fetal bovine serum (FBS), penicillin (100 U/ml), streptomycin (50 µg/ml), Lipo3000, and streptavidin agarose resin were from Thermo Fisher Scientific (Waltham, MA, USA). Tris [(1-benzyl-1H-1,2,3-triazol-4-yl) methyl] amine, tris (2-carboxyethyl) phosphine hydrochloride, triethylammonium bicarbonate buffer, urea, and 5-carboxy tetramethylrhodamine-azide were from Sigma-Aldrich (St. Louis, MO, USA). The total RNA extraction kit was from Omega (Norcross, GA, USA). The one-step ribonucleic acid (RNA) polymerase chain reaction (PCR) kit was from TaKaRa Bio (Kusatsu, Shiga, Japan). Avertin solution, Hoechst 33342/PI, and Cell Counting Kit-8 (CCK-8) were acquired from Solarbio (Beijing, China). The HDAC-Glo™ I/II Assay was from Promega (Madison, WI, USA).

### 4.2. Animals

Male C57BL/6J mice aged 6–8 weeks were obtained from Vital River, a Charles River Company, Beijing, China (http://www.vitalriver.com/, accessed on 1 June 2026.) (license: SCXK (Beijing) 2021-0006). All procedures were conducted according to a protocol approved by the Animal Care and Use Committee of the Institute of Chinese Materia Medica, China Academy of Chinese Medical Sciences (permit No.: 20192008), and performed in accordance with the U.K. Animals (Scientific Procedures) Act, 1986, and associated guidelines, EU Directive 2010/63/EU for animal experiments, and the National Research Council’s Guide for the Care and Use of Laboratory Animals. All animal protocols adhered to National Institute of Health requirements for activities involving the care and use of animals and were in compliance with the ARRIVE guidelines. Mice were housed in cages with a 12 h light/dark cycle in a room equipped with an air-filtering system maintaining 50–60% humidity at 23–25 °C. They were provided with standard maintenance food (Beijing, China, KEAO XIELI FEED CO., LTD. license: Beijing (2018) 06073) and ad libitum access to sterilized water.

The researchers tasked with allocation, treatment delivery, outcome evaluation, and data interpretation were oblivious to the group allocations until the conclusion of the final analysis. We used humane endpoints for animals when they were showing signs of moribund conditions including hypothermia and 20% weight loss. All efforts were made to minimize suffering and to reduce the number of animals used. We provided supplemental heat and massaged the animals suffering from acute renal injury, as well as checked the animal’s health every day. There were no unexpected deaths during our experiments.

### 4.3. Animal Experiments

AA-I, AA-IVa, and AL-I were each dissolved suspended in a 0.5% sodium carboxymethyl cellulose solution. Mice were randomly grouped using a random number table, intragastrically administered medium control or AAA, and designated as control (CTL), AA-I low dose (AA-I-L, 1 mg/kg), AA-I high dose (AA-I-H, 10 mg/kg), AA-IVa low dose (AA-IVa-L, 1 mg/kg), AA-IVa high dose (AA-IVa-H, 10 mg/kg), AL-I low dose (AL-I-L, 1 mg/kg), and AL-I high dose (AL-I-H, 10 mg/kg) groups. AAA was administered three times a week, every other day, for 24 weeks. Subsequently, some mice were euthanized with avertin solution (1.25%, sterile) and sacrificed immediately, and some were sacrificed at 45 weeks after AAA administration. Drug dose and duration were set according to the toxicity evaluation of other AAAs [2,6,47]. No animals or data points were excluded from the final analysis.

### 4.4. Histopathological Assessment

The fixed tissues were embedded in paraffin, cut into 2.5 μm thick sections, and stained with hematoxylin and eosin (HE), Masson’s trichrome, or immunohistochemistry (IHC) for morphological and protein expression evaluation. The histological alterations observed in the slides were blindly scored. Renal tubular damage (casts, pyknosis/karyolysis, and inflammatory infiltration) was semi-quantitatively evaluated (0–4) using HE staining. The scoring system was delineated as follows: a score of 0 signified no injury, characterized by normal tubular architecture and the absence of casts, pyknosis/karyolysis, or inflammatory infiltration. A score of 1 denoted mild injury (<25% involvement), marked by occasional casts, sporadic tubular epithelial cell pyknosis or karyolysis, and infrequent inflammatory cell infiltration. A score of 2 represented moderate injury (25–50% involvement), featuring relatively extensive cast formation, pronounced tubular epithelial cell pyknosis/karyolysis, and focal inflammatory infiltration. A score of 3 indicated severe injury (50–75% involvement), characterized by numerous casts, widespread tubular epithelial cell necrosis (pyknosis and karyolysis), and diffuse inflammatory cell infiltration. A score of 4 denoted very severe injury (>75% involvement), with significant disruption of tubular structure, extensive necrosis, cast formation, and dense inflammatory infiltration in nearly all examined fields [48]. The renal interstitial fibrosis was semi-quantitatively graded (0–4), and the collagen area was calculated by Masson’s trichrome staining. Grade 0 (no or mild fibrosis), fibrosis area ≤ 5%. Grade 1 (mild fibrosis), fibrosis area 6–25%. Grade 2 (moderate fibrosis), fibrosis area 26–50%. Grade 3 (severe fibrosis), fibrosis area 51–75%. Grade 4 (extremely severe fibrosis), fibrosis area > 75% [49]. IHC was conducted to assess the expression of α-SMA, TGF-β, HDAC2, NOTCH3, SPHK2, MYC, Caspase-7, BCL2, and BAX, utilizing semi-quantitative methodology grounded on mean optical density. In each part, five random fields of view at 200× magnification were chosen, and the photos were processed with Image-Pro Plus 6.0 software.

### 4.5. Cell Culture and Intervention

HK-2 cells were obtained from Bena Culture Collection (Xinyang, China). The cells were cultured in DMEM/F12 (1:1) supplemented with 10% FBS, penicillin (100 U/ml), and streptomycin (50 µg/ml) in an incubator with a humidified atmosphere of 5% CO_2_ and 95% air at 37 °C. Cells were treated with AA-I, AA-IVa, or AL-I at final concentrations of 20 μM or 40 μM for 24 h or 48 h; then, cells were collected for follow-up testing. Drug dose and duration were set according to the toxicity evaluation of AA-I [30]. To achieve HDAC2 knockdown, HK-2 cells were transfected with HDAC2-specific siRNA (siHDAC2) or negative control siRNA (NC) by Lipo3000 for 24 h before drug administration. The sense of siHDAC2 is 5′-GAGAUUUAAUGUUGGAGAA-3′; the antisense is 5′-UUCUCCAACAUUAAAUCUC-3′.

### 4.6. Transcriptome Sequencing

HK-2 cells (2.5 × 10^6^) were inoculated in T75 flasks, were permitted to adhere overnight, and thereafter underwent treatment. HK-2 cells were subjected to treatment with 40 μM AA-I, AA-IVa, AL-I or medium control (CTL) for 48 h, followed by RNA extraction for Illumina paired-end sequencing. Differential expression analysis was conducted with DESeq2 (|log2 FC (fold change)| > 1, *p* adj < 0.05), with CTL serving as the reference. Gene Ontology—Biological Process (GO-BP) and Kyoto Encyclopedia of Genes and Genomes (KEGG) enrichment analyses were conducted utilizing Metascape as previously [50]. Gene sets associated with kidney fibrosis from the HPO and DISGENT databases were gathered and analyzed for relationships with the differentially expressed genes (DEGs) discovered in the AAA-treated groups.

### 4.7. ABPP-CC and Molecular Docking

An alkyne group was introduced at the carboxyl position of AA-I to design the probe, designated as Probe 1. Modification at the carboxyl group may be superior to that at the nitrophenanthrene ring [51], as it not only improves the stability and selectivity of the probe but also better preserves the native structure and biological activity of AA-I. The utility of the probe was verified by a CCK-8 assay after manufacture. Target identification was performed by treating Probe 1 with total protein extracted from HK-2 cells, followed by biotin labeling by click chemistry. Biotinylated proteins were concentrated utilizing streptavidin agarose resin and identified through label-free mass spectrometry. GO-BP enrichment analysis was conducted on the discovered proteins, concentrating on gene sets associated with kidney fibrosis from the HPO and DISGENT databases.

The HDAC2 (PDB ID: 6WBZ) protein structure was sourced from the PDB database, while the ligand structures of AA-I (PubChem CID:2236), AA-IVa (PubChem CID: 161218), and AL-I (PubChem CID: 96710) were acquired from PubChem. Molecular docking was executed utilizing AutoDock Vina 4.2.6, while interaction analysis and visualization were conducted with PLIP and PyMOL 2.3.4.

### 4.8. High-Content Imaging Analysis and CCK-8 Assay

HK-2 cells (1 × 10^4^ per well) were inoculated in 96-well plates and permitted to adhere overnight, thereafter undergoing high-content imaging or CCK-8 assay. For high-content imaging, cells were exposed to AA-I, AA-IVa, or AL-I and subsequently stained with Hoechst 33342/PI. Images were captured using an Operetta system (6 fields per well, 200× magnification), and data were analyzed with Harmony 3.5.2 software to determine the percentage of apoptotic/necrotic cells in relation to the total cell count.

In the CCK-8 assay, for the Prode 1 availability experiment, HK-2 cells were exposed to complete medium containing Probe 1 or AA-I for 24 h (431.0–6.7 μM). In a distinct experiment, HK-2 cells were transfected with siHDAC2 and subsequently subjected to treatment AA-I (498.4–7.8 μM), AA-IVa (1008.3–15.8 μM), or AL-I (256.3–4.0 μM) for 48 h. Each group consisted of six replicate wells, and the experiment was conducted three times. After 24 h or 48 h, 10 μL of CCK-8 solution was introduced to each well, followed by incubation at 37 °C for 2 h. The absorbance at 450 nm was quantified.

### 4.9. Protein Purification and Surface Plasmon Resonance (SPR) Binding Assays

A human HDAC2 (Uniprot Accession No. Q92769) insect expression vector was engineered for protein purification. pFastBac1 was linearized using double digestion, and the HDAC2 gene underwent four rounds of PCR to incorporate a Twin-Strep tag at the C-terminus and to amplify the fragment. Following Gibson assembly, the construct was introduced into *E. coli* DH10Bac competent cells, resulting in the HDAC2 bacmid. The bacmid was introduced into SF9 cells obtained from Absea (Beijing, China). High-titer virus was amplified and utilized to reinfect SF9 cells for protein production. The target protein was isolated via Strep-tag affinity chromatography, resulting in recombinant HDAC2. Protein purity was evaluated using SDS-PAGE and Western blotting with Strep-HRP incubation.

SPR was conducted utilizing 1× PBS at pH 7.4, supplemented with 0.05% P20 and 1% DMSO as the running buffer. HDAC2 was affixed to a CM5 chip by amine coupling. AA-I, AA-IVa, and AL-I were serially diluted in running buffer and evaluated in a multi-cycle mode (association: 60 s; dissociation: 120 s). Data were evaluated with Biacore Insight Evaluation Software 4.0 with multi-cycle kinetics, conforming to a 1:1 binding model to compute the values of the association rate constants (ka), the dissociation rate constants (kd), and the equilibrium dissociation constants (KD).

### 4.10. Enzyme Activity Assay

HDAC2 enzyme activity was assayed according to the protocol provided by the manufacturer. Briefly, HDAC2 protein was respectively incubated with AA-I (1953.44–30.52 nM), AA-IVa (1958.36–30.60 nM), and AL-I (1953.41–30.52 nM), followed by the addition of a detection reagent containing an acetylated luminous peptide substrate, culminating in incubation and luminescence signal measurement. Emax and EC_50_ were calculated, with EC_50_ defined as the concentration producing 50% of Emax (top-to-bottom range).

### 4.11. Quantitative Polymerase Chain Reaction

HK-2 cell RNA was extracted and then real-time quantitative PCR was conducted to measure the mRNA expression levels of *TGF-β*, *ACTA2*, *Caspase-7*, *BCL2*, and *BAX*, utilizing *GAPDH* as an internal control. Relative gene expression was determined via the 2^−ΔΔ^Ct methodology [52]. *GAPDH* was purchased from Sangon Biotech (Shanghai, China). The primer sequences used are listed in Table S1.

### 4.12. Western Blot

Total protein was collected from lysates of HK-2 cells. Identical quantities of protein were resolved by SDS-PAGE and subsequently transferred to PVDF membranes. The membranes were incubated overnight at 4 °C with primary antibodies targeting TGF-β, HDAC2, MYC, Caspase-7, BCL2, and BAX, and then treated with secondary antibodies at room temperature. Blots were generated, images were captured with the Tannon 4800 Multi, and gray values were assessed with Image J 1.54f, employing GAPDH as an internal control.

### 4.13. Immunofluorescence (IF)

Cells were fixed with formalin, blocked with BSA, and incubated with primary antibodies targeting α-SMA, TGF-β, HDAC2, and MYC, followed by the application of fluorescent secondary antibodies. Nuclei were counterstained with DAPI. Coverslips were affixed and scanned utilizing a laser scanning confocal microscope. Five non-overlapping fields of view (200× magnification) were randomly selected for each coverslip, and the fluorescence intensity or the count of positive cells was assessed using ImagePro Plus software.

### 4.14. Statistical Analysis

All data are presented as means ± SD. One-way ANOVA was used to determine significant differences between multiple groups. The degree of pathological injury was analyzed using the Kruskal–Wallis test. The results were considered statistically significant at *p* < 0.05.

## 5. Conclusions

The toxicity of AAAs, including AA-I, AA-IVa, and AL-I, is varied; AA-I specifically targets HDAC2 to promote apoptosis and fibrosis, while AL-I shows reduced toxicity, and AA-IVa exhibits no apparent toxicity. This study provides new insights into the more prudent comprehension of herbal medicines (e.g., Xixin) that are rich in low-toxicity AAAs, offering a certain basis for their more rational governance and application in the future.

## Data Availability

The data presented in this study are available on request from the corresponding author due to the fact that a portion of the data is related to ongoing unpublished studies.

## Supplementary Materials

The following supporting information can be downloaded at: https://www.mdpi.com/article/10.3390/ph19101612/s1, Figure S1. Toxicity evaluation of AAAs including AA-I, AA-IVa, and AL-I. Figure S2. Purification and identification of human recombinant HDAC2 (h-HDAC2) protein. Table S1. Primer list for qPCR.

## Abbreviations

Xixin	ASARI RADIX ET RHIZOMA
AA-I	Aristolochic acid I
AA-IVa	Aristolochic acid IVa
AL-I	Aristolactam I
AAAs	Aristolochic acid analogs
AAs	Aristolochic acids
HDAC2	Histone deacetylase 2
HE	Hematoxylin and eosin
IHC	Immunohistochemistry
HK-2	Human renal tubular epithelial cells
IF	Immunofluorescence
qPCR	Quantitative polymerase chain reaction
SPR	Surface plasmon resonance
ABPP-CC	Activity-based protein profiling combined with click chemistry
CHP	The 2025 edition of the Chinese Pharmacopoeia
IARC	International Agency for Research on Cancer
AAN	Aristolochic acid nephropathy
UC	Urothelial carcinoma
KEGG	Kyoto Encyclopedia of Genes and Genomes
GO-BP	Gene Ontology—Biological Process
CCK-8	Cell Counting Kit-8
Prode 1	AA-I small-molecule probe
CTL	Control
