# Supplementary material for: HDAC2 Partially Contributes to the Differential Nephrotoxicity Induced by AA-I, AA-IVa, or AL-I
Source: Pharmaceuticals (Basel). 2026 Oct 10;19(10):1612. doi: 10.3390/ph19101612 (PMC13708075; doi:10.3390/ph19101612)
Supplement: Supplementary file 1 [file pharmaceuticals-19-01612-s001.zip › pharmaceuticals-4505932-supplementary.pdf]

Supplemental data

HDAC2 Partially Contributes to the Differential Toxicity of Aristolochic Acid Analogues including AA-I, AA-IVa, and AL-I

Yuan Wang[1, 2], Xian Zhong[1], Jing Meng[1], Chunying Li[1], Yong Zhao[1], Yan Yi[1], Jingzhuo Tian[1], Xiaoxin Gao[3], Chen Pan[1], Jiayin Han[1], Yushi Zhang[1], Suyan Liu[1], Chenyue Liu[1], Xingwen Wang[1], Feng Gao[1], Yuan Yu[4], Shuang Cui[1], Meiting Liu[1], Lianmei Wang[1*], Aihua Liang[1*]

[1]State Key Laboratory for Quality Ensurance and Sustainable Use of Dao-di Herbs, Beijing Key Laboratory of Psychoactive Substances Discovery and Control in Chinese Herbal Medicines, Key Laboratory of TCM Safety Risk Assessment and Translation, Institute of Chinese Materia Medica, China Academy of Chinese Medical Sciences, Beijing 100700, China

[2]Institute of Traditional Chinese Medicine, Tianjin University of Traditional Chinese Medicine, Tianjin 301617, China

[3]Beijing Institute for Drug Control, Beijing 102206, China

[4]College of Pharmacy, Guizhou College of Traditional Chinese Medicine, Guiyang 550025, China

* Correspondence: ahliang@icmm.ac.cn (A.L.); lmwang@icmm.ac.cn (L.W.)

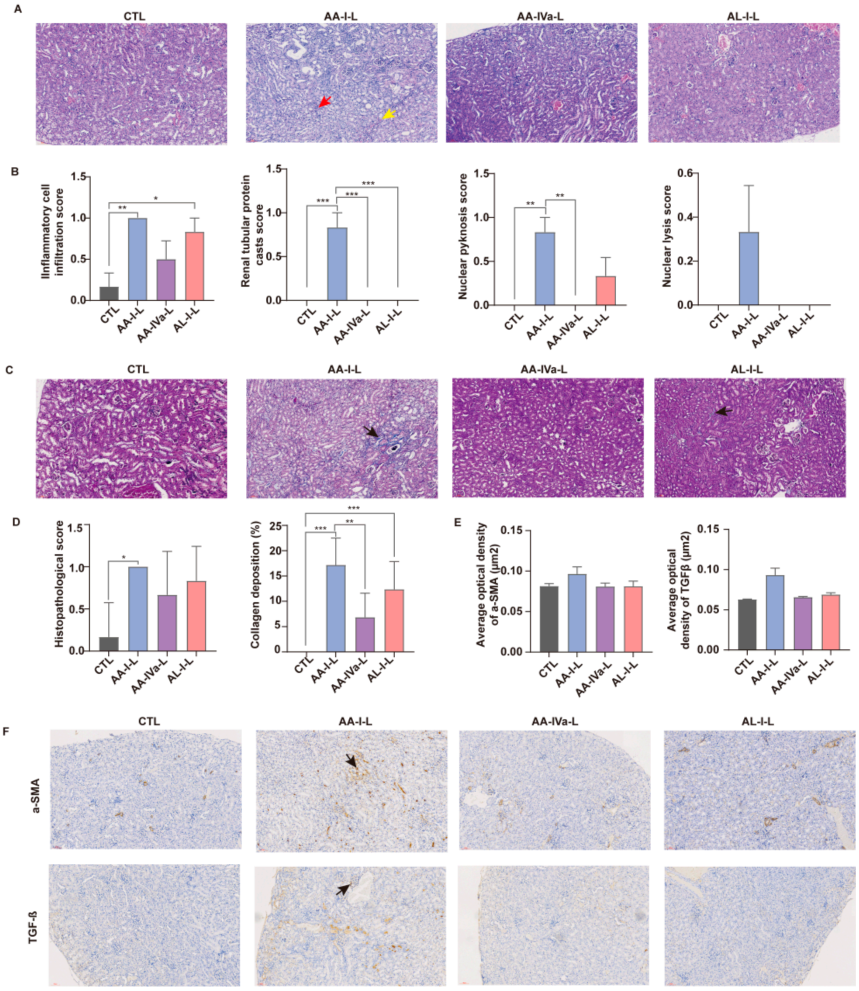

Figure S1. Toxicity evaluation of AAAs including AA-I, AA-IVa, and AL-I. (**A**,**B**) HE staining (**A**) and histological assessments (**B**) of mouse renal tissues subjected to 1 mg/kg AAAs treatment. In (**A**), yellow arrowheads indicate inflammatory cell infiltration, nuclear pyknosis, and karyolysis; red arrows indicate cast formation. (**C**,**D**) Masson's trichrome staining (**C**), fibrosis assessments, and quantitative (**D**) investigation of collagen deposition in mouse renal tissues subjected to 1 mg/kg AAAs treatment. In (**C**), arrowheads indicate renal fibrosis. (**E**,**F**) Semiquantitative (**E**) and IHC (**F**) assessment of renal fibrosis markers α-SMA and TGF-β in mouse renal tissues. In (**F**), arrowheads indicate IHC positive staining. *$P < 0.05$, ** $P < 0.01$, *** $P < 0.001$. $n = 6$.

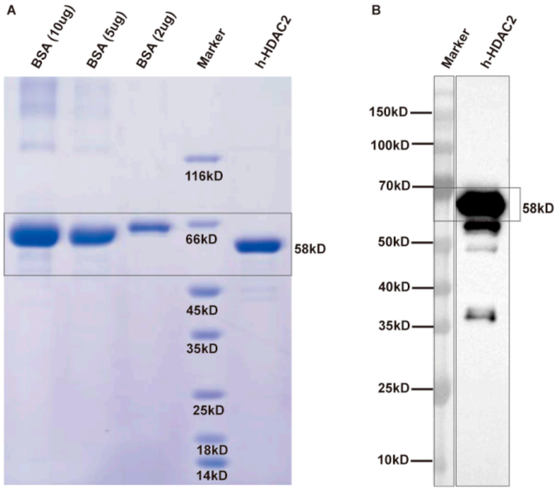

Figure S2. Purification and identification of human recombinant HDAC2 (h-HDAC2) protein. (**A**) Purity evaluation via SDS-PAGE staining with Comas-blue. (**B**) Western blot detection of h-HDAC2.

Table S1 Primer list for qPCR

Gene	Forward	Reverse	GenBank accession
TGF-β	5’-CTAATGGTGGAAACCC ACAACG-3’	5’-TATCGCCAGGAATTGTTGC TG-3’	NM_000660
ACTA2	5’-TATCGCCAGGAATTGTT GCTG-3’	5’-GCCATGTTCTATCGGGTAC TTC-3’	NM_001613
BCL2	5’-GCCATGTTCTATCGGG TACTTC-3'	5’-CGGTTCAGGTACTCAGTC ATCC-3’	NM_000657
BAX	5’-CCCGAGAGGTCTTTTT CCGAG-3’	5’-CCAGCCCATGATGGTTCT GAT-3’	NM_138763
Caspase-7	5’-CGGTCCTCGTTTGTA CCGTC-3’	5’-CGCCCATACCTGTCACTT TATCA-3’	NM_033339
